# Identification of genes, pathways and transcription factor-miRNA-target gene networks and experimental verification in venous thromboembolism

**DOI:** 10.1038/s41598-021-95909-4

**Published:** 2021-08-11

**Authors:** Yiming Su, Qiyi Li, Zhiyong Zheng, Xiaomin Wei, Peiyong Hou

**Affiliations:** Department of Vascular Surgery, LiuzhouWorker’s Hospital, Fourth Affiliated Hospital of Guangxi Medical University, Liuzhou, 545005 Guangxi Province China

**Keywords:** Cardiovascular biology, Computational biology and bioinformatics, Molecular biology, Biomarkers

## Abstract

Venous thromboembolism (VTE) is a complex, multifactorial life-threatening disease that involves vascular endothelial cell (VEC) dysfunction. However, the exact pathogenesis and underlying mechanisms of VTE are not completely clear. The aim of this study was to identify the core genes and pathways in VECs that are involved in the development and progression of unprovoked VTE (uVTE). The microarray dataset GSE118259 was downloaded from the Gene Expression Omnibus database, and 341 up-regulated and 8 down-regulated genes were identified in the VTE patients relative to the healthy controls, including CREB1, HIF1α, CBL, ILK, ESM1 and the ribosomal protein family genes. The protein–protein interaction (PPI) network and the transcription factor (TF)-miRNA-target gene network were constructed with these differentially expressed genes (DEGs), and visualized using Cytoscape software 3.6.1. Eighty-nine miRNAs were predicted as the targeting miRNAs of the DEGs, and 197 TFs were predicted as regulators of these miRNAs. In addition, 237 node genes and 4 modules were identified in the PPI network. The significantly enriched pathways included metabolic, cell adhesion, cell proliferation and cellular response to growth factor stimulus pathways. CREB1 was a differentially expressed TF in the TF-miRNA-target gene network, which regulated six miRNA-target gene pairs. The up-regulation of ESM1, HIF1α and CREB1 was confirmed at the mRNA and protein level in the plasma of uVTE patients. Taken together, ESM1, HIF1α and the CREB1-miRNA-target genes axis play potential mechanistic roles in uVTE development.

## Introduction

Venous thromboembolism (VTE) refers to a condition wherein blood clots are formed in the vasculature that block the blood reflux, leading to deep venous thrombosis (DVT) and pulmonary embolism (PE)^1^. The annual incidence of VTE ranges from 75/100,000 to 136/100,000 in the Western countries, and 10.6% and 23% of these patients within 30 days or 1 year respectively^2,3^. In Hong Kong, the annual incidence rate is 41.7/100,000 and the 30-day mortality rate is 39.7%^4^. Approximately 0.1–9% of the PE patients develop chronic thromboembolic pulmonary hypertension and almost 50% of the DVT patients progress to post-thrombotic syndrome within 3 months^5^. Studies increasingly show that vascular injury and vascular endothelial damage are the risk factors of VTE in both adult and pediatric populations^6–8^. Endothelial cells (ECs) form the vascular intima that lines all blood vessels. Healthy ECs release anti-thrombotic factors such as thrombomodulin and tissue plasminogen activator, whereas injured ECs release coagulants like the von Willebrand factor^9^. In addition, adhesion between leukocytes and the activated endothelium in injured vessels may activate the coagulation cascade and lead to platelet accumulation^10^. Although there is evidence of a link between endothelial dysfunction and the initiation and progression of VTE^11^, the underlying molecular mechanisms and signaling pathways are poorly understood.

Micro RNAs (miRNAs) are a class of small, non-coding RNAs consisting of 18 to 23 nucleotides that regulate target gene expression at the post-transcriptional level, either by blocking target mRNA translation or by promoting its degradation. The miRNA-encoding genes are first transcribed into primary miRNAs by RNA polymerase II, which are subsequently processed into pre-miRNAs by a nuclear protein complex called microprocessor. The pre-miRNAs are cleaved by dicer and loaded into argonaute protein, resulting in the formation of mature miRNAs and the RNA-induced silencing complex that regulates the expression of target genes. However, the regulation of miRNAs biogenesis and function is highly complex, and some miRNAs are generated by drosha-independent or dicer-independent mechanisms. In addition, multiple factors affect miRNAs processing, stability and function, such as post-translational modification of argonaute proteins and changes in the miRNA sequence^12,13^. Studies increasingly show that miRNAs are involved in the pathogenesis of cardiovascular diseases, and are potential diagnostic biomarkers and therapeutic targets^14,15^. A recent study reported a substantial number of differentially expressed plasma miRNAs between VTE patients and healthy controls^16^. For example, miR-424-5p and miRNA-320a/b were up-regulated in VTE patients, and their respective area under the curve (AUC) in the receiver-operating characteristic (ROC) curve analysis for diagnosing VTE were 0.62, 0.70 and 0.79^17,18^. In addition, miR-145 is known to reduce thrombogenesis by targeting the coagulation factor XI *in vivo*^19^, and some miRNAs influence the viability of endothelial progenitor cells in DVT patients and enhance thrombus recanalization and resolution^20^. However, the potential role of miRNAs in the vascular ECs (VECs) of patients with VTE needs further investigation.

Transcription factors (TFs) regulate gene expression by binding to the target gene promotor sequences adjacent to transcription initiation sites or distal enhancer elements. Recent developments in whole-genome sequencing and functional genomics have identified TFs associated with various physiological and pathological processes, including cardiovascular diseases^21^ and VTE. For example, hypoxia-inducible factor (HIF)1α, a TF targeting vascular endothelial growth factor (VEGF)^22^, promoted angiogenic factor production and accelerated thrombus resolution and vein recanalization in a murine inferior vena cava model^23^. In addition, HIF1α can promote the initiation or development of VTE by enhancing pro-inflammatory and innate immune responses via VEGF and serpin family E member 1 (PAI-1)^24^. These studies indicate that HIF1α plays an important role in maintaining the balance between thrombolysis and thrombosis. The cAMP response element binding protein (CREB) also drives thrombospondin-1 transcription, and is involved in the formation and maintenance of arterial thrombosis^25,26^. Wang et al. showed that the TF Yin Yang (YY)1 up-regulated vitronectin expression by binding to the rs2227721-G (rs is an id of dbSNP database and G is a base located at rs222721) variant of its promoter. The G to T mutation in rs2227721 decreased the binding efficiency between YYI and vitronectin promoter and increased risk of DVT, indicating that YY1 is a repressor of thrombosis^27^. Recent studies also show that TFs can regulate gene expression by binding to the promoter of cognate miRNAs^28^. A potential role of TFs and the TF-miRNAs axis in the VECs of VTE patients has not been reported so far.

We conducted bioinformatics analysis to identify the differentially expressed genes (DEGs) and pathways in the VECs of patients with unprovoked VTE (uVTE) relative to healthy controls. Furthermore, we also predicted potential miRNAs and TFs regulating the VTE-associated genes and pathways. In addition, the expression levels of some potentially crucial genes were confirmed at the mRNA and protein levels in plasma of uVTE patients with routine molecular biology assays. Our findings provide novel insights into targeted VTE therapy.

## Methods

### Ethics statement

Ethics approval was waived for microarray expression profiling since publicly available datasets were used for the analysis. Data acquisition and application were as per the guidelines of the gene expression omnibus (GEO) database. All human studies were approved by the Ethics Committee of Liuzhou Worker’s Hospital, Fourth Affiliated Hospital of Guangxi Medical University (Approval Number: 20180518), and conducted according to the relevant guidelines and regulations. Written informed consent was obtained from all subjects.

### Study population

A total of 12 patients with uVTE and 12 age- and sex-matched healthy volunteers were enrolled (Supplementary Information-Table S1). All patients had VTE diagnosed as acute, symptomatic DVT of the lower extremities. All uVTE had been objectively confirmed by D-dimer and color doppler examination. Fasting blood samples were collected from all participants and then centrifuged at 3000×*g* for 10 min at 4 °C. Serum samples were then collected and stored at − 80 °C for qRT-PCR analysis and ELISA.

### Identification of DEGs and hierarchical clustering analysis

**Figure 1:**
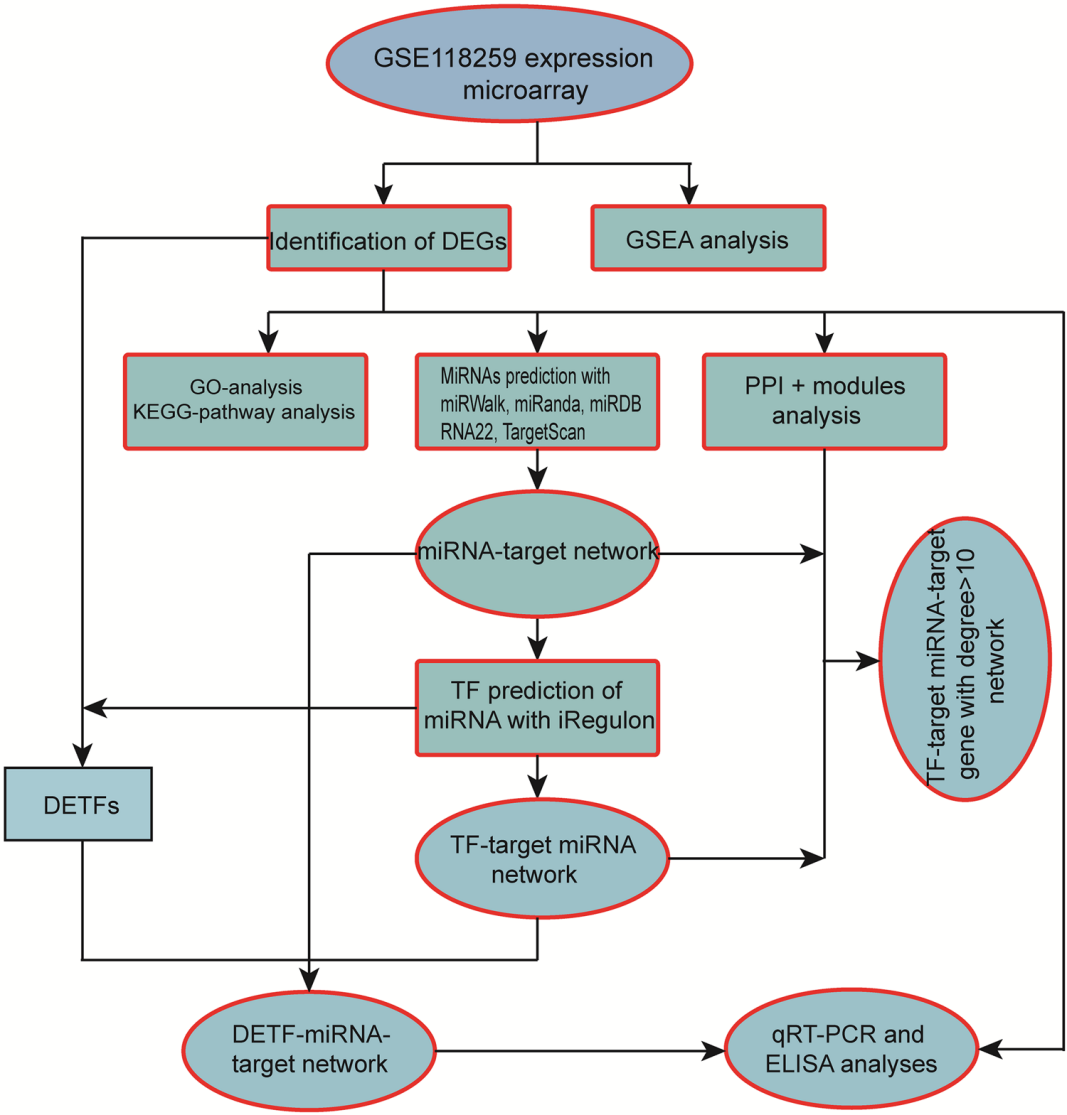
Flow diagram of the analysis procedure.

The workflow of this study is shown in Fig. 1. The microarray dataset GSE118259 based on the GPL10558 Platform (Illumina HumanHT-12 V4.0 expression beadchip) was downloaded from the GEO database (https://www.ncbi.nlm.nih.gov/geo/, accessed November, 2019). It includes data from a total of 13 endothelial colony-forming cell samples isolated from uVTE patients (n = 8) and healthy controls (n = 5). The DEGs between the datasets were screened using the GEO2R online tool with |logFC|> 1 and adj.p value < 0.05 as the cut-off criteria. A volcano plot was constructed and hierarchical clustering analysis was performed using the ggplot2 package and the pheatmap package in R respectively.

### Functional and pathway enrichment analyses

The Database for Annotation, Visualization and Integrated Discovery version 6.8 (DAVID; https://david.ncifcrf.gov/, accessed November, 2019) was used for functional classification and annotation of the DEGs^29,30^. Gene Ontology (GO), and the Kyoto Encyclopedia of Genes and Genomes (KEGG) and REACTOME pathway enrichment analyses were performed. Homo Sapiens was selected as species and background and *p* values < 0.05 were considered statistically significant. Gene set enrichment analysis (GSEA) was performed to explore potential mechanisms based on the Molecular Signatures Database (MSigDB) including the c2 (c2. cp. kegg. V7.4. symbol. gmt and c2. cp. wikipathway. V7.4. symbol. gmt) and c5 (c5. go. bp or cc or mf V7.4. symbol. gmt) (http://software.broadinstitute.org). The background of GSEA was Illumina HumanHT-12 V4.0 expression beadchip. The number of permutations was set at 1,000 and nominal *P*-value < 0.05 and false discovery rate (FDR) < 0.25 were considered statistically significant.

### PPI network of DEGs and module analysis

The Search Tool for the Retrieval Interacting Genes (STRING) version 11.0 (https://www.string-db.org/, accessed December, 2019) was used to analyze the interaction of protein-encoding genes^31–33^. All DEGs were uploaded to the STRING online tool to construct the PPI network, which was then visualized using Cytoscape software 3.6.1. “Homo sapiens” and an interaction score > 0.4 (medium confidence) were the parameters. Module analysis of the PPI network was performed with the MOCDE app of Cytoscape software 3.6.1. The parameters for MCODE analysis were MCODE score > 3.5 and the number of nodes > 10.

### Construction of the miRNA-target DEG regulatory network

The miRNAs targeting the DEGs were predicted using the miRWalk1.0 (http://zmf.umm.uni-heidelberg.de/apps/zmf/mirwalk/, accessed December, 2019)^34^, miRanda (http://www.microrna.org/microrna/home.do, accessed December, 2019)^35^, miRDB (http://www.mirdb.org/, accessed December, 2019)^36^, RNA22 (https://cm.jefferson.edu/rna22/, accessed December, 2019)^37^ and TargetScan (http://www.targetscan.org/vert_72/, accessed December, 2019) databases^38^. The miRNA- argeting DEG pairs predicted by all five databases were imported into the Cytoscape software 3.6.1 to construct the miRNA-target DEG regulatory network.

### Construction of the TF‑miRNA‑target gene regulatory network

The TFs regulating miRNAs were predicted using the iRegulon plug-in (http://apps.cytoscape.org/ apps/iRegulon) of the Cytoscape software 3.6.1, and the Transfac (http://www.gene-regulation.com/pub/databases.html, accessed January, 2020) and Encode (https://www.encodeproject.org/, accessed January, 2020) databases^39,40^. An enrichment score threshold > 3, a minimum identity of 0.0 between orthologous genes, and a maximum false discovery rate (FDR) of 0.001 on motif similarity were the thresholds. The predicted TFs that were also identified as DEGs were designated differentially expressed transcription factors (DETFs), and used to construct the DETF‑miRNA‑target gene network. In addition, the protein-encoding DEGs with a degree of more than 10 in the PPI network were selected to build the TF-miRNA-target gene network in accordance with the miRNA-target DEG and the TF-miRNA pairs. All networks were built and visualized using Cytoscape software 3.6.1.

### RNA extraction and qRT-PCR analysis

Total RNA was isolated from serum samples using TRIzol according to the manufacturer’s instructions (Invitrogen, Carlsbad, California, USA). For each sample, the total RNA content was determined by measuring the absorbance at 260 nm and the purity was ascertained in terms of A260/A280. First strand cDNAs of the miRNAs and mRNAs were synthesized using the Mir-X miRNA first strand synthesis kit and PrimeScript RT reagent kit with gDNA eraser (Takara Biotechnology, Dalian, China) respectively. RT-PCR was performed using 2 µl of each cDNA template and the FastStart SYBR Green Kit (Roche, East Sussex, UK) on the ABI 7500 Fast cycler (Applied Biosystems, Darmstadt, Germany)^41^. The primer sequences are shown in Supplementary Information-Table S2.

### ELISA

The serum levels of gene expression were measured using the appropriate ELISA kits (Solarbio Life Sciences, Beijing, China). Optical density (OD) was measured at 450 nm according to the manufacturer’s instruction.

### Statistical analysis

SPSS statistical software for Windows, version 22.0 (SPSS, Chicago, IL, USA), was used for statistical analysis. Groups were compared using t-test and *P* < 0.05 was considered statistically significant. Data were shown as mean ± standard deviation (SD).

## Results

### Screening of DEGs and hierarchical clustering analysis

**Figure 2:**
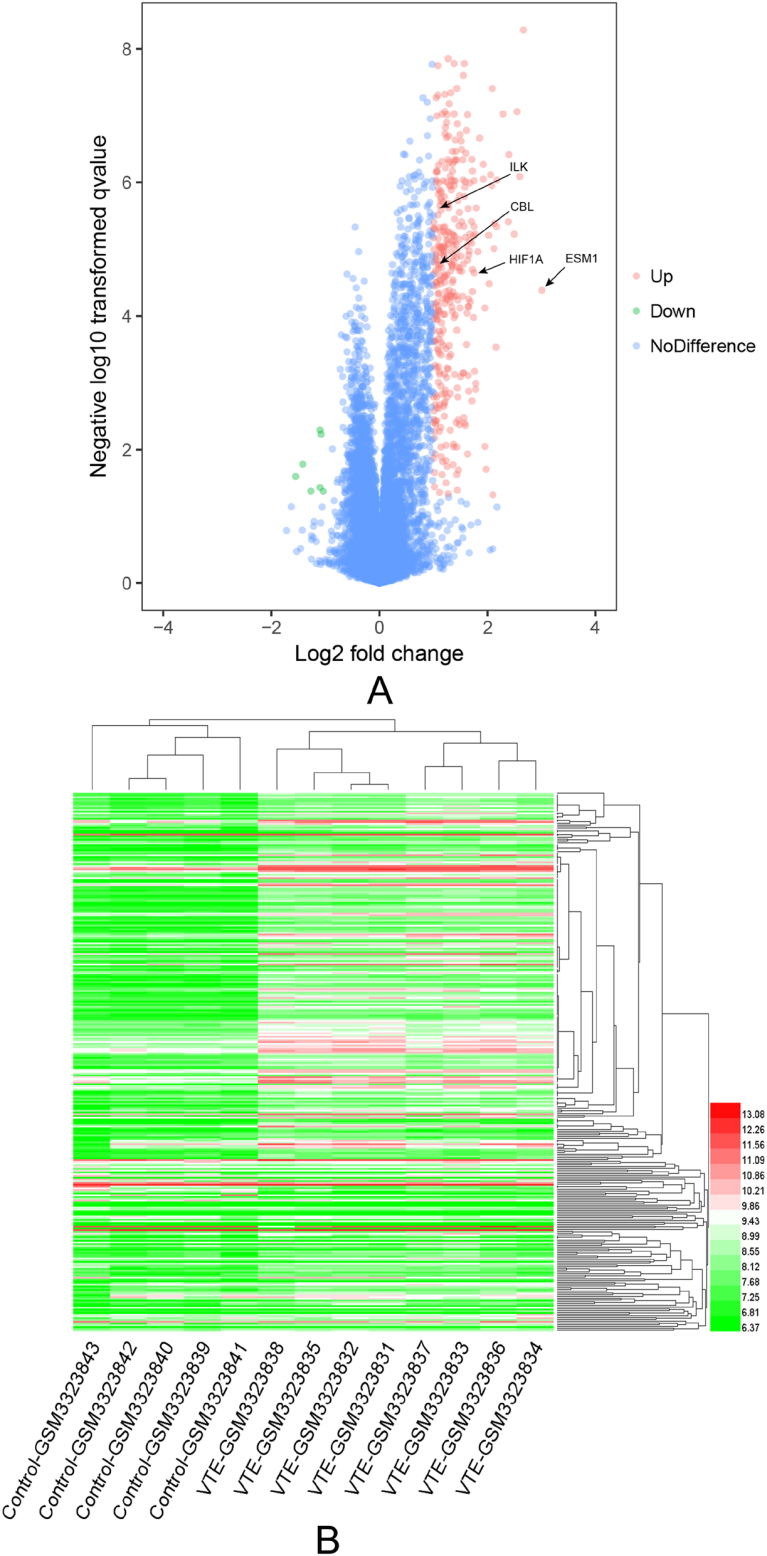
Identification of DEGs and hierarchical clustering analysis. (**A**) The volcano plot of GSE118259, with red nodes indicating up-regulated genes with logFC > 1 and p value < 0.05, green nodes down-regulated genes with logFC < -1 and p value < 0.05, and blue nodes similarly expressed genes. (**B**) Heat map of DEGs. The bottom horizontal axis shows sample names and the top horizontal axis shows the sample clusters. Each row represents a single gene and the right vertical axis represents DEG clusters. Colors closer to red indicate high gene expression levels and those closer to green indicate low gene expression levels. Differentially expressed genes, DEGs, Fold change, FC. Created with R soft 3.5.3 (https://mirrors.tuna.tsinghua.edu.cn/CRAN/).

Based on the cutoff criteria of |logFold change| (logFC) > 1 and adj.p value < 0.05, a total of 341 up-regulated and 8 down-regulated endothelial colony-forming cell-specific genes were identified between the patient and control datasets. *HIF1α*, *CBL*, *ILK* and *ESM1* had a higher fold change, as shown in the volcano plot (Fig. 2A) and in Supplementary Information-Table S3. The hierarchy cluster analysis clearly showed that the DEGs can distinguish between the VTE patients and control group (Fig. 2B). These results suggest that HIF1α, CBL, ILK and ESM1 play possibly an important role in VTE.

### Functional and pathway enrichment analysis

**Figure 3:**
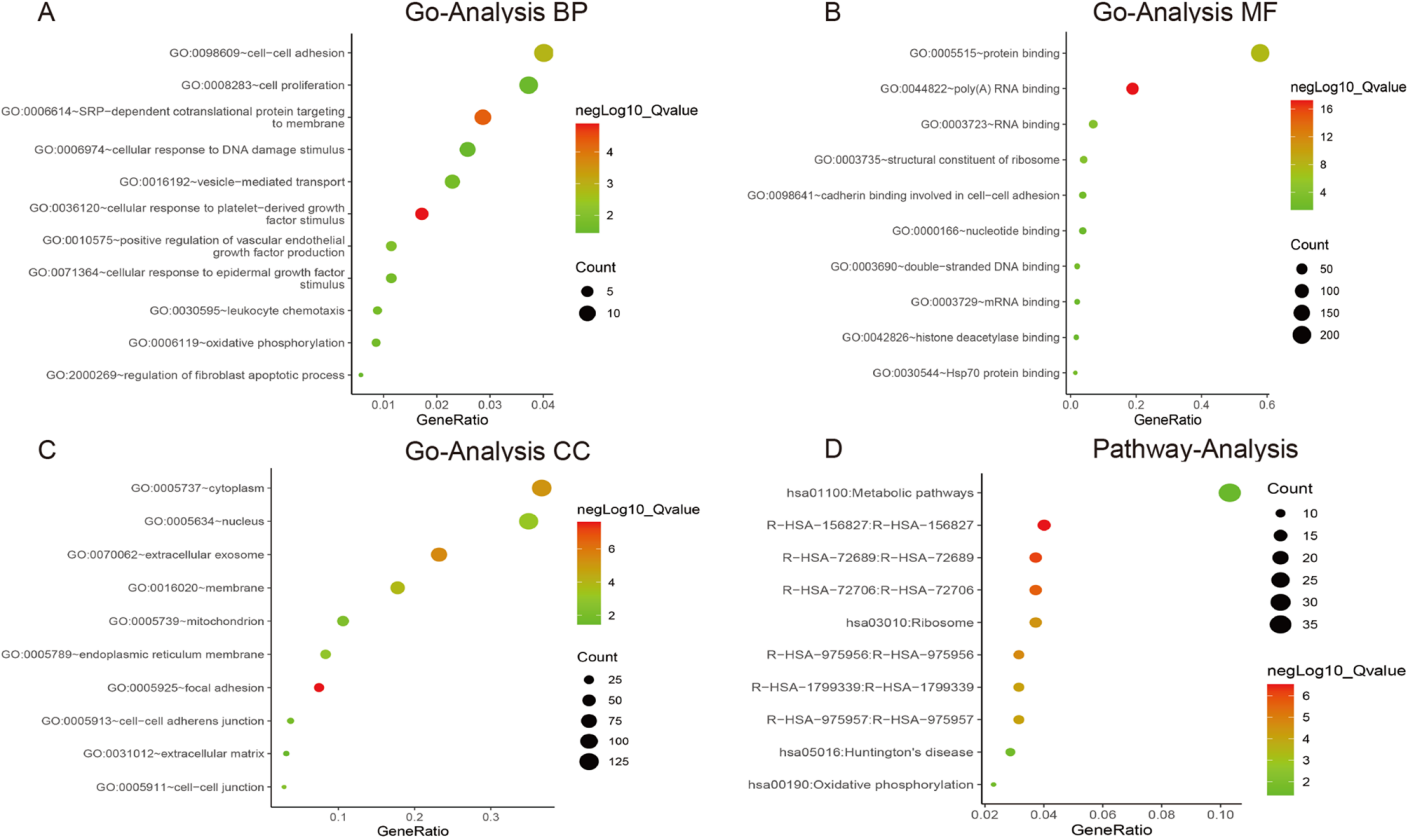
GO and pathway enrichment analyses of DEGs. (**A–D**) represent significant GO Biological Process (BP), GO Molecular Function (MF) and GO Cellular Component (CC) terms, and enriched pathways of the DEGs respectively. Differentially expressed genes, DEGs, Gene ontology, GO. Created with DAVID version 6.8 (https://david.ncifcrf.gov/, accessed November, 2019) and R soft 3.5.3 (https://mirrors.tuna.tsinghua.edu.cn/CRAN/).

**Figure 4:**
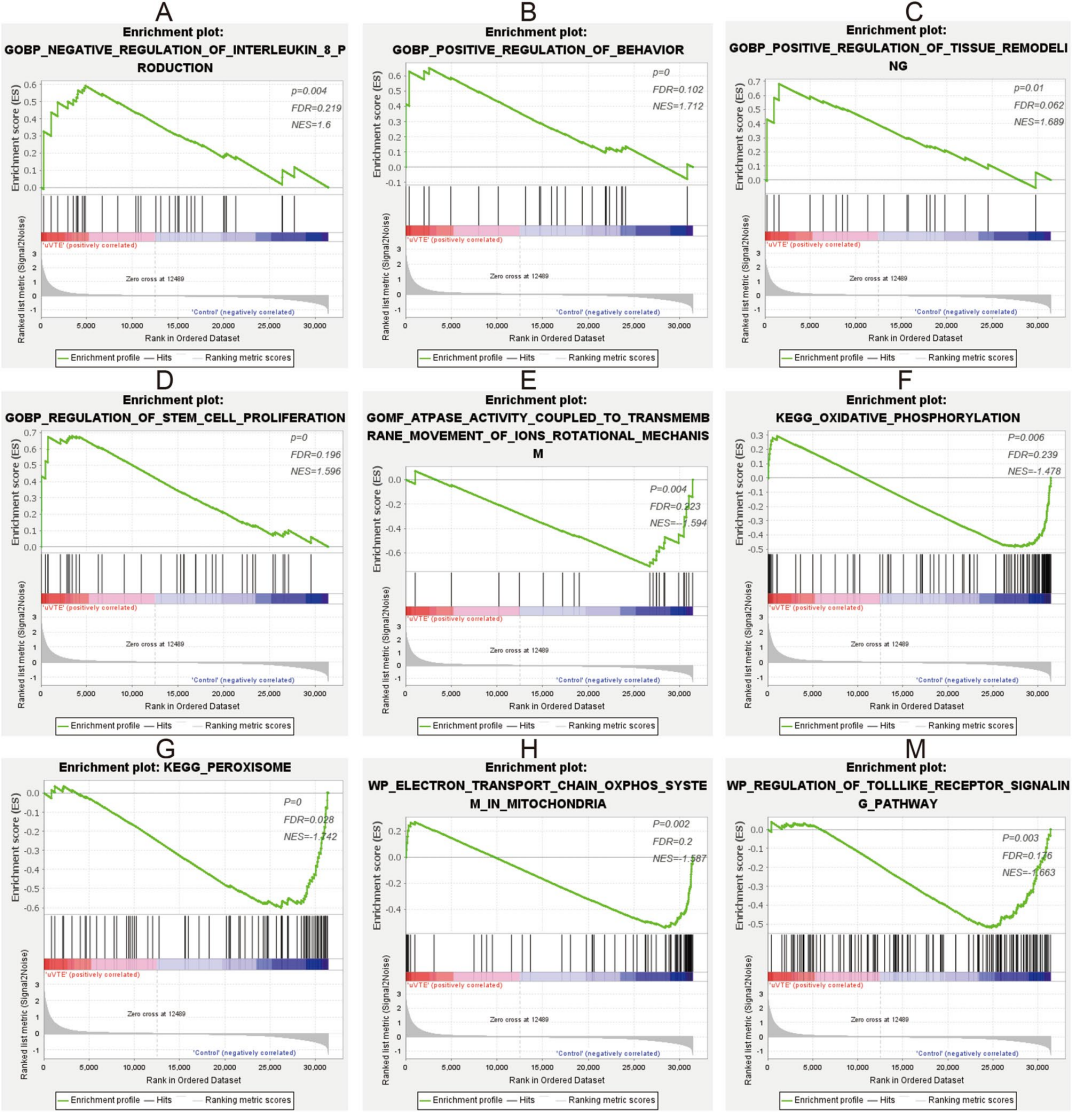
GSEA results of uVTE and control group samples. (**A–E**) GSEA results of reference gene sets regarding GO terms in c5 GO V7.4. symbol. gmt. (**F–M**) GSEA results of reference gene sets regarding pathways in c2 KEGG V7.4. symbol. gmt and c2 wikipathway V7.4. symbol. gmt. *GSEA* gene set enrichment analysis, *NES* normalized enrichment score, *uVTE* unprovoked Venous Thromboembolism, *KEGG* Kyoto Encyclopedia of Genes and Genomes, *Go* Ontology, GO. Created with GSEA soft_4.1.0 (http://www.gsea-msigdb.org/gsea/downloads.jsp).

To further elucidate the biological changes in uVTE, the DEGs were annotated for the GO biological process (BP), GO cellular component (CC) and GO molecular function (MF) terms. The significant GO BP terms were cell–cell adhesion (GO:0098609), cell proliferation (GO:0008283), cellular response to DNA damage stimulus (GO:0006974), SRP-dependent cotranslational protein targeting to membrane (GO:0006614), oxidative phosphorylation (GO:0006119), positive regulation of vascular endothelial growth factor production ( GO:0010575) and cellular response to platelet-derived growth factor stimulus and epidermal growth factor stimulus (GO:0036120 and GO:0071364). For the GO CC terms, the DEGs were mainly enriched in focal adhesion (GO:0005925), cell–cell junction (GO:0005911), cell–cell adherens junction (GO:0005913), cytoplasm (GO:0005737), nucleus (GO:0005634) and extracellular exosome (GO:0070062). The main GO MF terms were cadherin binding involved in cell–cell adhesion (GO:0098641), protein binding (GO:0005515), poly(A) RNA binding (GO:0044822), structural constituent of ribosome (GO:0003735) and nucleotide binding (GO:0000166). In addition, metabolic pathways (hsa01100) including metabolism of protein (R-HSA-156827, R-HSA-72689 and R-HSA-72706), metabolism of RNA (R-HSA-975957), Ribosome (hsa03010) and oxidative phosphorylation (hsa00190) were highly enriched among the DEGs (Fig. 3 and Supplementary Information-Table S4). As shown in the GSEA results, the DEGs were significantly enriched in oxidative phosphorylation, peroxisome, positive regulation of behavior, positive regulation of tissue remodeling, negative regulation of interleukin 8 production, cell proliferation, regulation of toll like receptor signaling pathway (Fig. 4 and Table Supplementary Information-Table S5). Cell adhesion was significantly enriched in multiple GO analyses. Given that CBL, ILK, ESM1 were also enriched in cell adhesion, they may regulate VTE progression by influencing the biological behavior of endothelial cells.

### PPI network and Modular Analysis

**Figure 5:**
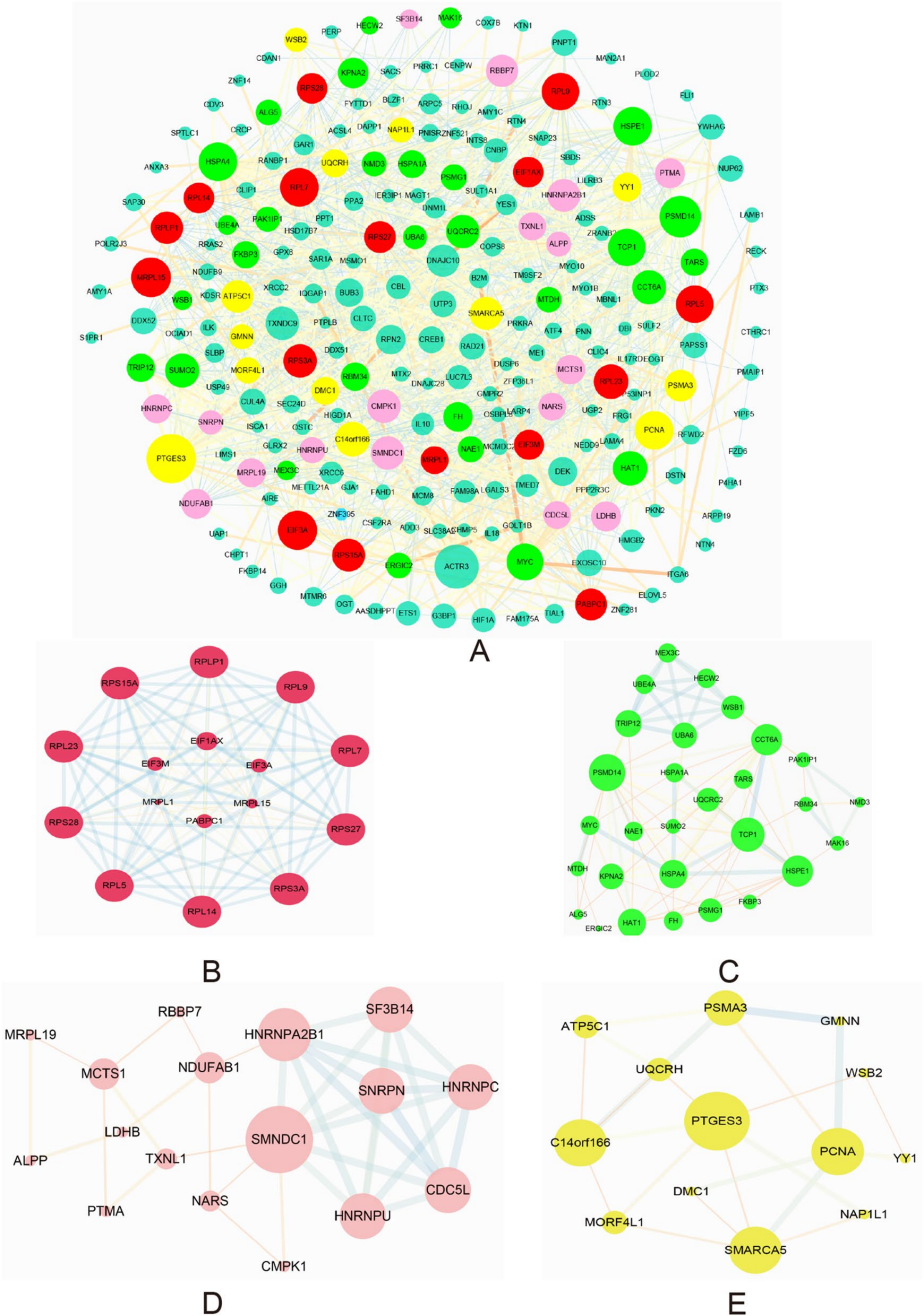
PPI network of the DEGs and module analysis. (**A**) PPI network constructed using 237 protein-encoding DEGs and 1105 edges. Node size indicates node degree, and the red, green, pink and yellow nodes represent module 1, module 2, module 3 and module 4 genes respectively. (**B**) Module 1 consists of 16 nodes and 113 edges. (**C**) Module 2 consists of 29 nodes and 90 edges. (**D**) Module 3 consists of 17 nodes and 36 edges. (**E**) Module 4 consists of 13 nodes and 22 edges. In module analysis, node size indicates node degree, edge size and edge color based on combined score. Protein–protein interaction, PPI, Differentially expressed genes, DEGs. Created with STRING soft version 11.0 (https://string-db.org) and cytoscape software 3.6.1 (www.cytoscape.org).

The PPI network was constructed to evaluate the relationship between the protein-encoding DEGs, and consisted of 1105 edges corresponding to 237 protein-encoding DEGs. Only ZNF395 was down-regulated, while the remaining 236 DEGs (Fig. 5A) including HIF1α, ILK, CBL and CREB1 were upregulated, of which only CREB1 had a degree ≥ 10. In addition, module analysis using Cytoscape MCODE enriched four modules from the PPI network based on the degree of importance. Module 1 mainly consisted of the ribosomal protein family and 113 edges (Fig. 5B) that were related to the metabolism of proteins, metabolism of RNAs, translational initiation, focal adhesion and binding GO BP terms (Table 1 and Supplementary Information-Table S6). Module 2 contained 29 nodes and 90 edges (Fig. 5C), which were involved in the GO BP terms of poly (A) RNA binding, protein binding and ATP binding (Table 1 and Supplementary Information-Table S6). Module 3 comprised of 17 nodes and 36 edges (Fig. 5D), which were mainly enriched in the GO BP terms of mRNA splicing and RNA binding (Table 1 and Supplementary Information-Table S6), while module 4 was made up of 13 nodes and 22 edges (Fig. 5E) that participated in protein binding and cell proliferation (Table 1 and Supplementary Information-Table S6). These results further indicated that HIF1α, ILK, CBL and CREB1 may play a key role in VTE and and endothelial cell metabolism.

**Table 1:** Pathway enrichment analysis of Module genes function.

Module	Term	Count	*P*
Module1	R-HSA-156827: L13a-mediated translational silencing of Ceruloplasmin expression	14	9.87E-24
	R-HSA-72689: Formation of a pool of free 40S subunits	13	1.18E-21
	R-HSA-72706: GTP hydrolysis and joining of the 60S ribosomal subunit	13	4.19E-21
	R-HSA-975957: Nonsense Mediated Decay (NMD) independent of the Exon Junction Complex (EJC)	11	2.03E-16
	GO:0003723 ~ RNA binding	10	1.55E-10
	GO:0005515 ~ protein binding	16	5.53E-05
	GO:0005925 ~ focal adhesion	7	3.99E-07
	GO:0006413 ~ translational initiation	14	4.10E-26
Module2	GO:0044822 ~ poly(A) RNA binding	11	7.43E-06
	GO:0005515 ~ protein binding	24	9.40E-04
	GO:0005524 ~ ATP binding	7	0.033050441
	hsa04120: Ubiquitin mediated proteolysis	3	0.034610994
Module3	GO:0000398 ~ mRNA splicing, via spliceosome	5	3.62E-05
	R-HSA-72163/mRNA Splicing—Major Pathway	6	1.12E-06
	GO:0003723 ~ RNA binding	5	0.001119369
Module4	GO:0005515 ~ protein binding	11	0.008427604
	GO:0008283 ~ cell proliferation	3	0.027066307
	GO:0006119 ~ oxidative phosphorylation	2	0.009253701

### The microRNA–target gene regulatory network

**Figure 6:**
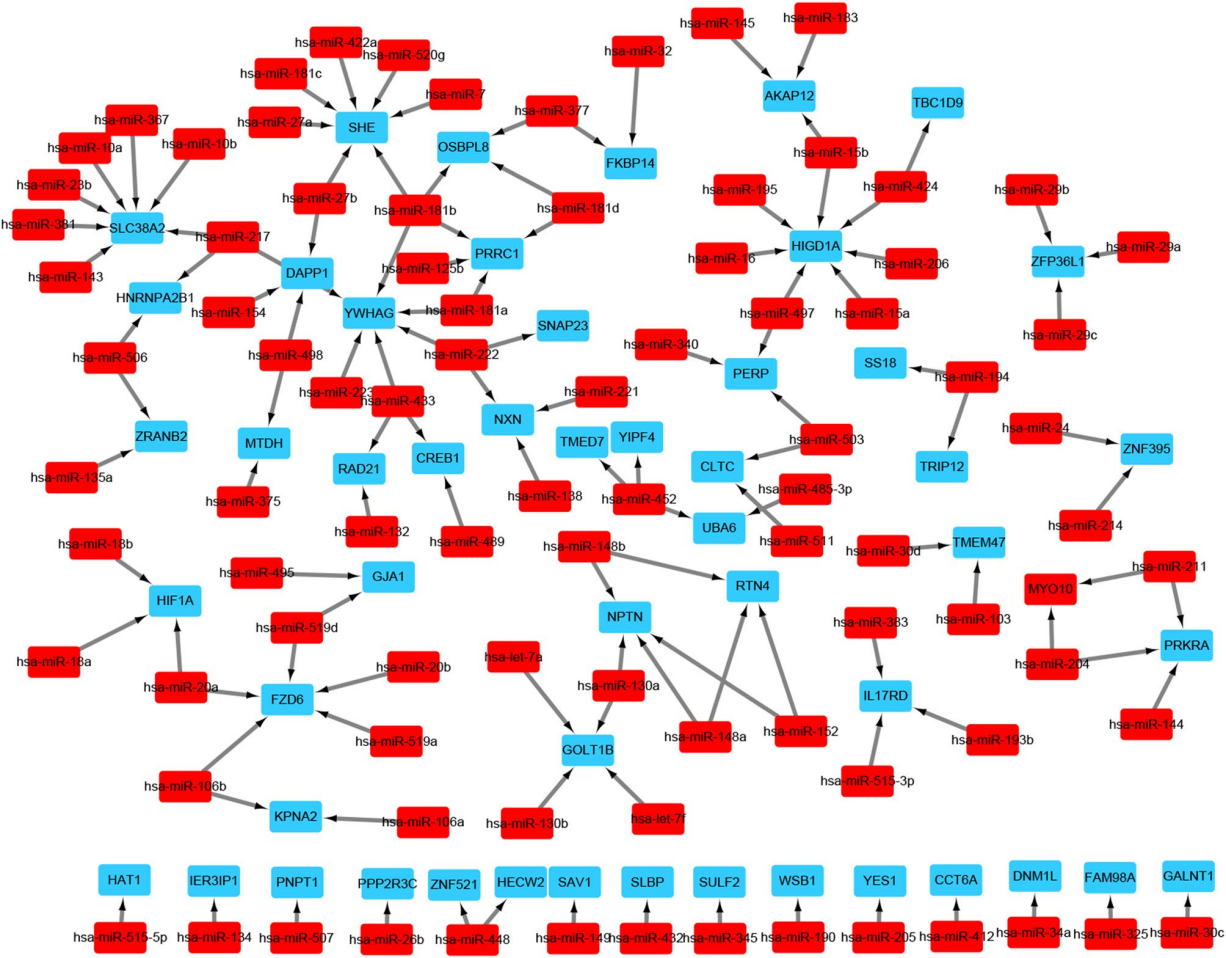
MiRNA-target DEGs interaction networks. Red nodes represent miRNAs and blue nodes represent target DEGs. Arrows indicate miRNA-target relationship. Differentially expressed genes, DEGs. Created with cytoscape software 3.6.1 (www.cytoscape.org).

To identify the potential miRNAs regulating initiation and progression of VTE, we screened 89 miRNAs from the miRWalk 1.0 database and 121 miRNA-DEG pairs were identified. Each miRNA-target DEG pair was simultaneously predicted by the miRanda, miRDB, miRWalk, RNA22 and TargetScan databases. The miRNA target DEG network consisted of 89 miRNAs, 52 DEGs and 121 edges, and included the has-miR-18b-HIF1α, has-miR-489-CREB1, has-miR-433-CREB1, has-miR-222-NXN, has-miR-222-SANP23, has-miR-222-YWHAG, has-miR-125b-PRRC1, has-miR-206-HIGD1A, has-miR-29b-ZFP36L1, has-miR-34a-DNM1L and has-miR-135a-ZRANB2 pairs. In addition, HIGD1A, SHE and SLC38A2 were putative hub targeting genes with seven different miRNAs, and hsa-miR-181b was the putative targeting miRNA for 4 DEGs (Fig. 6).

### TF‑miRNA‑target DEG regulatory network analysis

**Figure 7:**
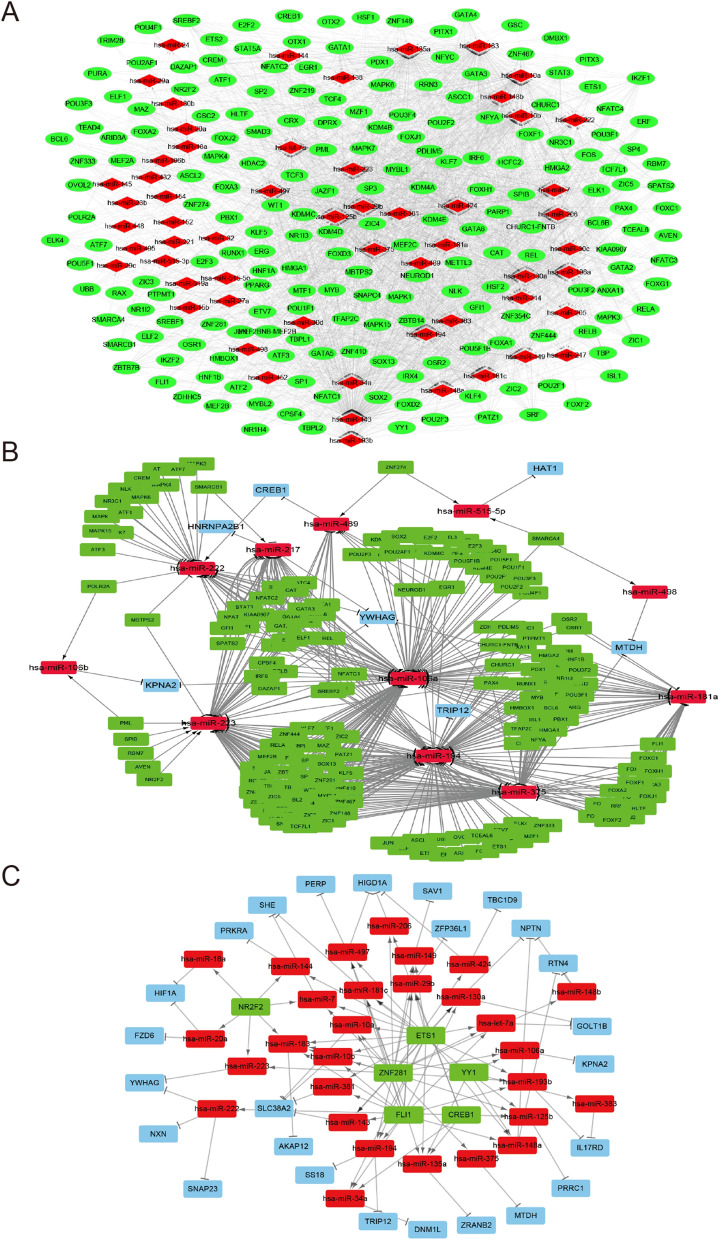
Transcription factor (TF) regulatory network. (**A**) TF-miRNA regulatory network. the red and green nodes indicate miRNAs and TFs, respectively. (**B**) TF-miRNA-target DEGs regulatory network. Blue nodes represent DEGs with a degree > 10 in the PPI network. (**C**) DETFs-miRNA-target regulatory network. Blue nodes represent DEGs, and the red and green nodes indicate miRNAs and DETFs respectively. The arrow indicates the TF-miRNA target relationship and the T shape represents the miRNA-target DEG relationship. TF, Transcription factor, DEGs, Differentially expressed genes, PPI, Protein–protein interaction, DETFs, Differentially expressed Transcription Factors. Created with cytoscape software 3.6.1 (www.cytoscape.org).

**Figure 8:**
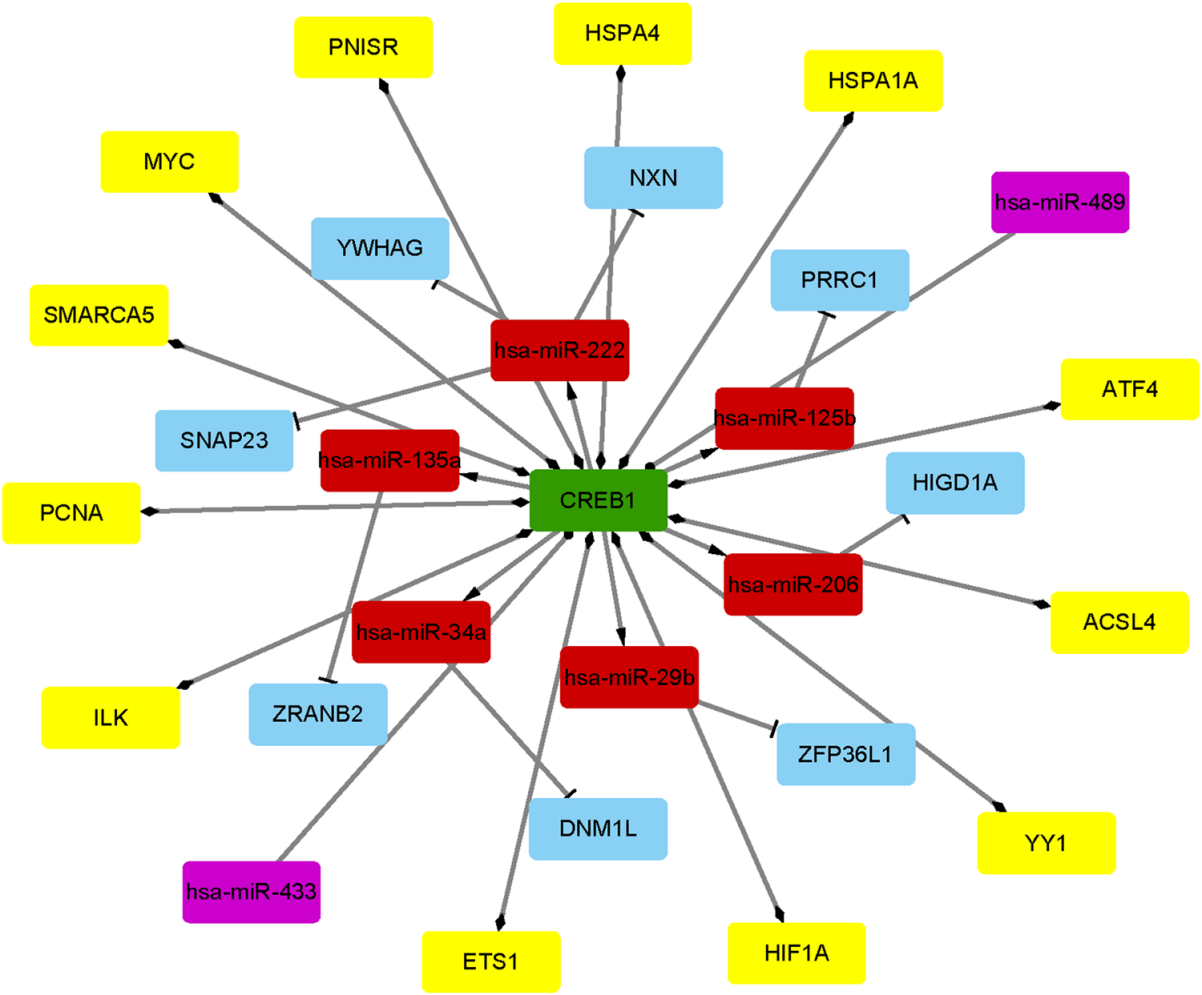
CREB1 regulatory network. Green node indicates CREB1. Red nodes indicate predicted target miRNAs of CREB1. Blue nodes represent target DEGs of miRNAs. Purple nodes represent predicted miRNAs regulating CREB1. Yellow nodes represent DEGs interacting with CREB1 in the PPI network. The arrow indicates the CREB1-miRNA target relationship and the T shape represents the miRNA-target relationship. The circle represents the miRNA-CREB1 target relationship and the diamond represents CREB1-DEGs relationship. Differentially expressed genes, DEGs, Protein–protein interaction, PPI. Created with cytoscape software 3.6.1 (www.cytoscape.org).

Based on the iRegulon app of Cytoscape software, a total of 197 TFs were predicted as regulators of miRNAs in the miRNA-target gene regulatory network, of which CREB1, ZNF281, NR2F2, ETS1, FLI1 and YY1 were the DETFs. The TF-miRNA regulatory network consisted of 2839 TF-miRNA pairs, 197 TFs and 58 miRNAs (Fig. 7A). CREB1 regulated 6 miRNAs–hsa-miR-34a, hsa-miR-29b, hsa-miR-222, hsa-miR-206, hsa-miR-135a and hsa-miR-125b – in the TF-miRNA network. Among the miRNA- DEG pairs, the protein-encoding DEGs with a degree ≥ 10 in the PPI network (CREB1, HIF1A, ILK, YY1, MYC etc.) were screened to construct the TF‑miRNA‑target DEG regulatory network. There were 12 miRNA-DEG pairs, 528 TF-miRNA pairs, 197 nodes and 540 edges in this network (Fig. 7B). CREB1 and other five DETFs were also used to construct a DETF‑miRNA‑target network, which consisted of 60 nodes, 111 edges, and the 6 CREB1-miRNA pairs and their corresponding 8 miRNA-target gene pairs (Fig. 7C). Finally, based on all the protein-coding DEGs and the miRNAs targeting and targeted by CREB1, a comprehensive CREB1 regulatory network was constructed (Fig. 8). The results indicated that some DEGs, such as CREB1, HIF1A etc., are associated with multiple regulatory networks in the progression of VTE.

### Potential crucial genes levels in plasma of uVTE patients

**Figure 9:**
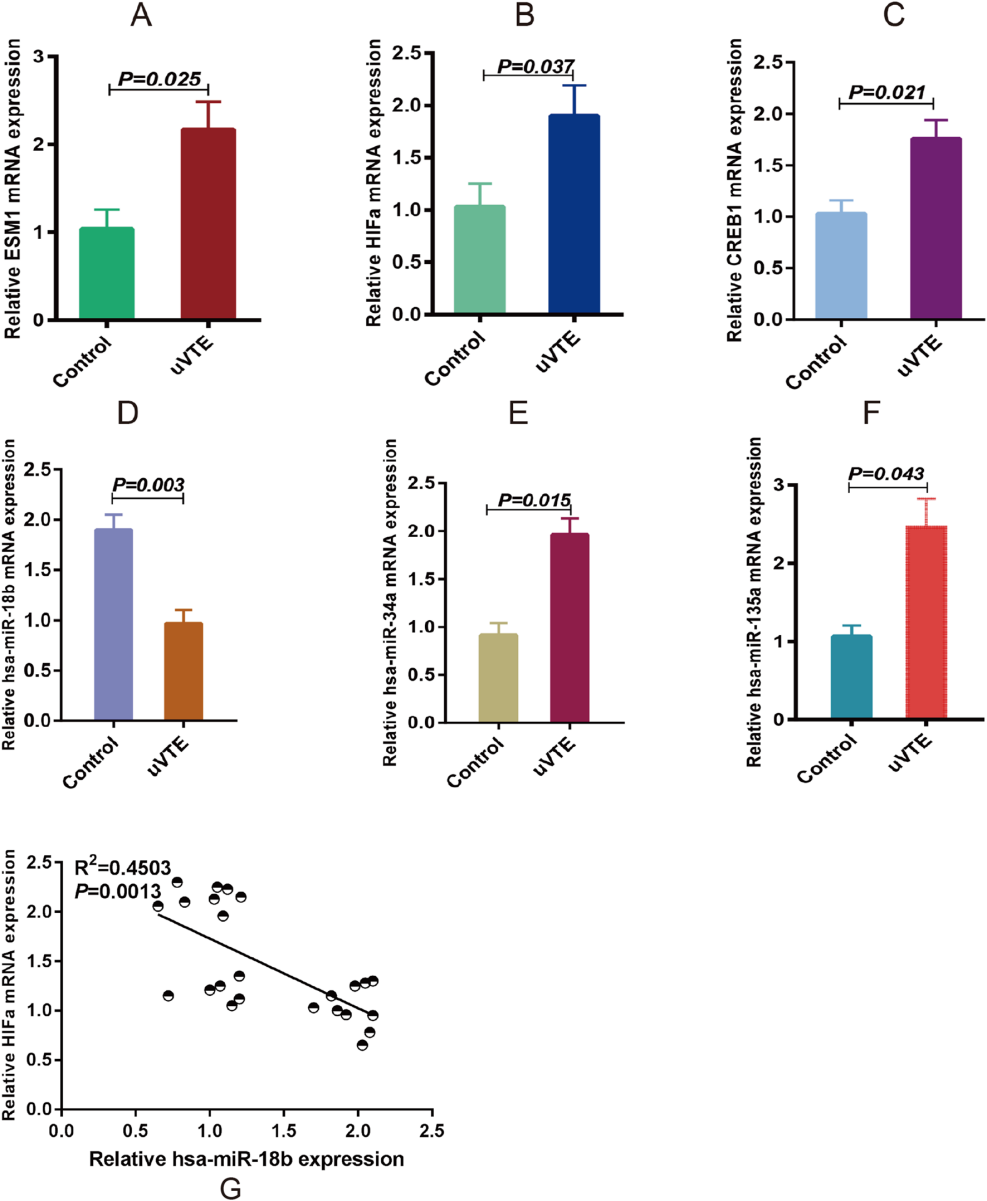
Expression levels of some crucial genes in the plasma of uVTE patients. (**A–E**) ESM1, HIF1α and CREB1 mRNA levels in the plasma of uVTE patients and healthy controls. (**D–F**) hsa-miR-18b, hsa-miR-34a, hsa-miR-135a levels in the plasma of uVTE patients and healthy controls. G, Correlation analysis between HIFa and has-miR-18b. Unprovoked Venous Thromboembolism, uVTE, quantitative Real-time- Polymerase Chain Reaction, qRT-PCR.

**Figure 10:**
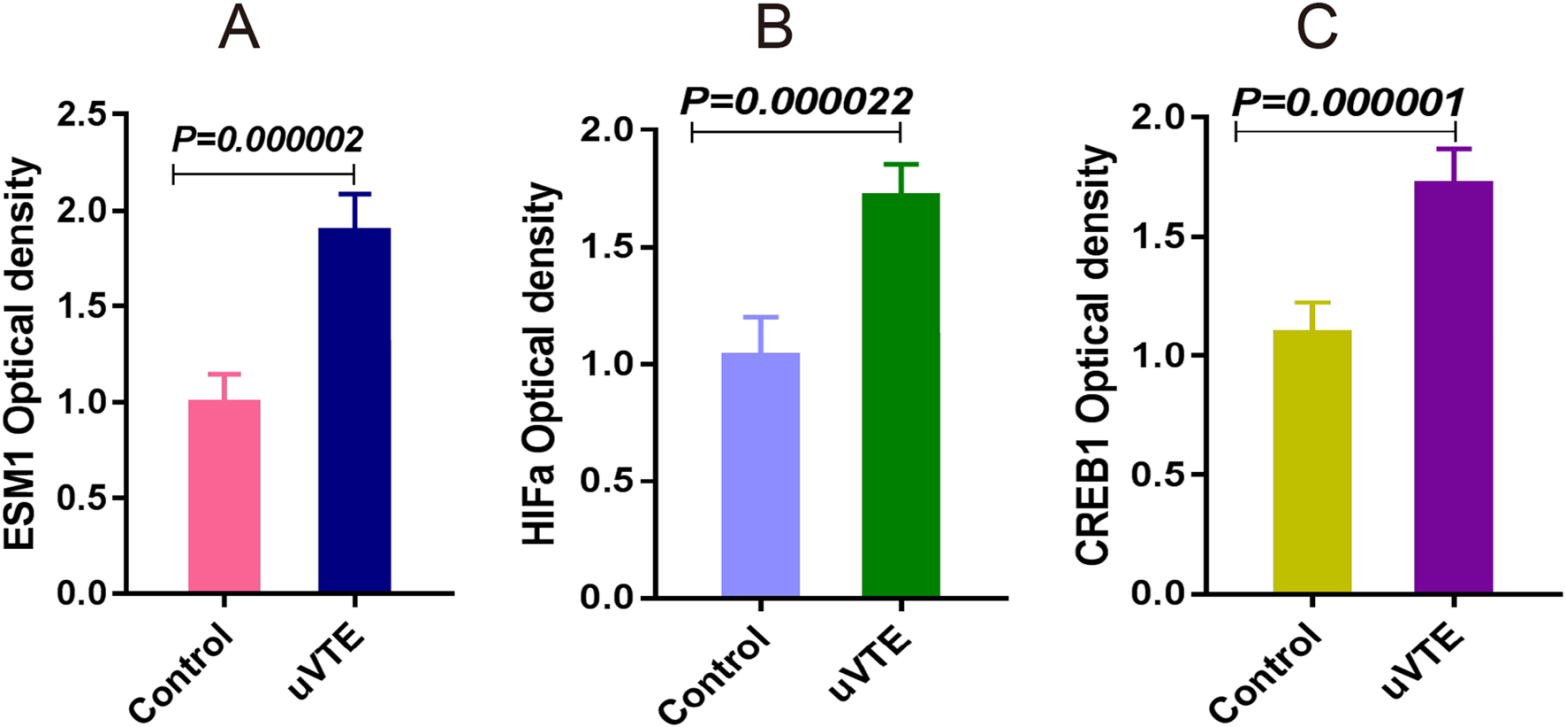
The proteins levels of some crucial genes in the plasma of uVTE patients. (**A–C**) ESM1, HIF1α and CREB1 protein levels in the plasma of uVTE patients and healthy controls. Unprovoked Venous Thromboembolism, uVTE, Enzyme Linked Immune Sorbent Assay, ELISA.

To further confirm the biological role of these identified DEGs in VTE, we measured the levels of ESM1, HIF1α, CBL, ILk, CREB1, hsa-miR-18b, hsa-miR-34a, hsa-miR-135a, hsa-miR-29b, has-miR-489 and has-miR-433 levels in the plasma samples of uVTE patients. As shown in Fig. 9, ESM1, HIF1α, CREB1, hsa-miR-34a and hsa-miR-135a were upregulated, whereas hsa-miR-18b was downregulated in the patient samples. Furthermore, has-miR-18b was negatively correlated with HIFa (Fig. 9). Consistent with this, the ESM1, HIF1α and CREB1 protein levels were also up-regulated in uVTE patients (Fig. 10 and Supplementary Information-Table S7). In contrast, CBL, ILk and other miRNAs were not statistically significant and may need further verification on a larger cohort. Taken together, ESM1, HIF1α and CREB1 are crucial genes involved in VTE progression.

## Discussion

We identified several putative uVTE-associated genes and pathways, along with one TF-miRNA-target gene axis, in human ECs. CREB1, ESM1, HIF1α, CBL, ILK and ribosomal protein family mRNAs were differentially expressed between the uVTE and control groups. In addition, ESM1, CBL, ILK, CREB1 and HIF1α were significantly enriched in biological processes of cell adhesion and cellular response to growth factor stimulus respectively. CREB1 was identified as a DETF involved in the TF-miRNA-target gene axis. The altered expression levels of ESM1, HIF1α, CREB1, hsa-miR-34a, hsa-miR-135a and hsa-miR-18b in uVTE was confirmed in patient plasma samples. Taken together, the aforementioned factors likely regulate the miRNA/target gene axis which activates EC adhesion and initiates VEC injury in uVTE.

Bella et al^42^ also found that the TNFSF15–TNFRSF25 axis is upregulated in uVTE and involved in endothelial dysfunction. Although the same microarray data was used in both studies, the results were different, which can be attributed to the distinct methodologies and aims. While Bella et al. focused on the genes related to the regulation of endothelial function, we applied systems biology to identify multiple crucial genes and pathways and construct a regulatory network.

Vascular endothelial damage is a major risk factor of thrombosis, and may disrupt the balance between coagulation and anticoagulant systems following decreased fibrinolysis^43^. In this study, ECs derived from VTE patients and healthy controls were analyzed, and a large number of DEGs were enriched in metabolism of protein, metabolism of RNA, mRNA transcription and translation, and cellular response to DNA damage stimulus pathways, as well as the GO term of cell proliferation. We hypothesize therefore that ECs increase the proliferative capacity of ECs after endothelial damage as a self-healing mechanism. In a stasis-induced rat DVT study, the majority of DEGs between the thrombosis and control groups were mainly expressed by ECs, and therefore enriched in EC-related functions. In addition, the top three enriched pathways or biological processes of DEGs were also related to metabolism and cell growth^44^. We also constructed a PPI network of the DEGs, and found that some genes in module 1 and module 3, for e.g. ribosomal proteins and thioredoxin like 1 (TXNL1), are associated with apoptosis^45,46^. However, some genes in module 2 and module 4 are associated with cell proliferation and cell migration, such as proteasome 26S subunit, non-ATPase 14 (PSMD14), MYC proto-oncogene and YY1^47–49^. These results indicate that altered EC viability may lead to vascular injury and even VTE.

Since neutrophils are the first cells to adhere to injured vessels via the activated endothelium^10,50^, changes in EC adhesion may be associated with thrombosis. Consistent with this, the DEGs in our study were enriched in GO terms like cell–cell adhesion, cell adhesion molecule binding and protein binding involved in cell adhesion, further underscoring their role in VTE. ESM1, CBL and ILK were enriched in the biological process of cell adhesion, and ESM1 was the top-ranked DEG in this study. Since it is an established predictor of sepsis severity^51^, ESM1 is also likely associated with the coagulation cascade. Mosevoll et al. reported that plasma ESM1 levels, in combination with plasma E-selectin, C-reactive protein and D-dimer, can be used to distinguish DVT^52^. Consistent with this, ESM1 mRNA and protein levels were significantly up-regulated in the plasma of uVTE patients. Therefore, we hypothesize that ESM1 participates in VTE by enhancing EC adhesion. CBL and ILK promote thrombosis by regulating platelet activation, and CBL increases platelet aggregation and thrombosis via αIIbβ3 and GPVI signaling^53,54^. ILK levels are increased in arterial thrombosis patients, and ILK-/- mice showed reduced platelet activation and subsequent aggregation, leading to unstable thrombosis^55,56^. Both CBL and ILK were upregulated in the uVTE patients in our study, albeit not significantly. A larger cohort may be required to further verify their expression levels in uVTE patients. Finally, factors involved in cell adhesion are significantly up-regulated in the plasma of VTE patients as well as mouse models^57–61^. Taken together, excessive EC adhesion is the major underlying mechanism of VTE, and is mediated by ESM1, CBL and ILK.

GO analysis also showed the enrichment of biological processes such as cellular response to platelet-derived growth factor (PDGF) stimulus, epidermal growth factor (EGF) stimulus and VEGF. Consistent with our findings, a previous study showed that the target genes of circulating miRNAs in DVT patients were enriched in the VEGF signaling pathway^62^. In addition, high-throughput affinity plasma proteomic profiling of VTE patients showed that PDGF-β was an independent risk factor of thrombosis-related disorders^63^, and HIF1α and CREB1 were enriched in growth factor related-GO BP terms with VEGF as the common target in murine macrophages^22,64^. VEGF is a risk factor of VTE, and regulates proliferation and migration of ECs. High levels of VEGF have been reported in cancer-related VTE^65,66^. It also induces tissue factor, the initiator of the coagulation cascade, and activates the fibrinolytic system by inducing the tissue-type plasminogen activator (tPA), urokinase-type plasminogen activator (uPA) and plasminogen activator inhibitor 1^67^. Interestingly, CREB and its coactivator protein p300 can maintain functional integrity of the HIF-1a/VEGF transcription machinery and subsequent angiogenesis^68^. In this study, we found that CREB1 and HIFa were also up-regulated in the plasma of uVTE patients. Taken together, enrichment of growth factor-related GO terms in the uVTE-associated DEGs are closely associated with endothelial dysfunction, and the CREB-HIF-1a/VEGF axis likely plays a crucial role in VTE.

In the PPI network, some genes in module 2 were associated with HIF1α, which promotes SOCS box containing 1 (WSB1) expression in hypoxic cells, indicating that module 2 mediates HIF-1 signaling pathway to initiate VTE^69^. In addition, HIF1α was also a predicted target of hsa-miR-18b and was negatively correlated with has-miR-18b in this study. Similar to our study, Dolt et al. found that the HIF1α mRNA 3'UTR is highly conserved across humans, mice and rats, which is indicative of its biological significance^70^. Wang et al. and Chen et al. found that overexpression or inhibition of miR-18b by mimics or inhibitors respectively can suppress or promote HIFα expression in normal human trophoblast cell lines and malignant melanoma cell lines. Furthermore, Chen et al. confirmed that HIFα is a direct target gene of miR-18b based on luciferase reporter assay. In addition, CREB1 was predicted as the targeting gene of has-miR-489 and has-miR-433, and 6 other miRNAs were predicted as CREB1 targets. Hsa-miR-34a and hsa-miR-135a were also up-regulated in the plasma of uVTE patients, whereas hsa-miR-18b was down-regulated. Several DEGs encoding ribosomal proteins were also identified and enriched in module 1. The ribosomal family proteins are upregulated in the plasma of VTE patients^71,72^, and are known to trigger venous thrombus formation in aged mice via p53 activation^73^. Furthermore, ribosomal proteins are also detected in circulating platelets and are associated with VTE^74,75^. Therefore, based on the above results, we hypothesize that the hsa-miR-18b/HIF1α/VEGF axis, the CREB1-miRNA-target genes axis and the ribosomal protein family genes are associated with VTE and need to be investigated further.

Our findings provide new insights into the pathogenesis of uVTE and identify potential diagnostic and therapeutic targets/pathways. However, further experimental studies and independent cohort studies are needed to validate these findings.

## Supplementary Information

Supplementary Legends.

Supplementary Table S1.

Supplementary Table S2.

Supplementary Table S3.

Supplementary Table S4.

Supplementary Table S5.

Supplementary Table S6.

Supplementary Table S7.

## Data Availability

The data used to support the findings of this study are available in the supplementary information.
