# Supplementary material for: Identification of genes, pathways and transcription factor-miRNA-target gene networks and experimental verification in venous thromboembolism
Source: Sci Rep. 2021 Aug 11;11:16352. doi: 10.1038/s41598-021-95909-4 (PMC8357955; doi:10.1038/s41598-021-95909-4)
Supplement: Supplementary file 1 — Supplementary Legends. [file 41598_2021_95909_MOESM1_ESM.docx]

**Identification of Genes, Pathways and Transcription factor-miRNA-target Gene Networks and Experimental Verification in Venous Thromboembolism**

**Yiming Su, Qiyi Li, Zhiyong Zheng, Xiaomin Wei, Peiyong Hou**

**Supplementary Information-Table Legends:**

**Supplementary Information-Table S1: Clinical features of the study population in this study.**

**Supplementary Information-Table S2: Primer sequences used for Real Time PCR**

**Supplementary Information-Table S3: Identification of differentially expressed genes in the GSE118259**

**Supplementary Information-Table S4: Gene ontology and pathway enrichment analyses of DEGs**

**Supplementary Information-Table S5:** **GSEA results of uVTE and control group samples**

**Supplementary Information-Table S6: Pathway enrichment analysis of Module genes function**

**Supplementary Information-Table S7: Optical density of ELISA trial**
