# Supplementary material for: Identification of genes, pathways and transcription factor-miRNA-target gene networks and experimental verification in venous thromboembolism
Source: Sci Rep. 2021 Aug 11;11:16352. doi: 10.1038/s41598-021-95909-4 (PMC8357955; doi:10.1038/s41598-021-95909-4)
Supplement: Supplementary file 3 — Supplementary Table S2. [file 41598_2021_95909_MOESM3_ESM.docx]

**Table S2 Primers used for Real Time PCR**

| Gene symbol | Forward | Reverse |
| --- | --- | --- |
| ESM1  HIF1a  CREB1  CBL  ILK  GAPDH  Has-miR-34a  Has-miR-135a  Has-miR-18b  Has-miR-29b  Has-miR-489  Has-miR-433  U6 | GGTGTCAGCCTTCTAATGGG  GAACGTCGAAAAGAAAAGTCTCG  TTAACCATGACCAATGCAGCA  GACAGTATGGGCTGGCAGTT  GACATTGTCGTGAAGGTGCTGAA  TCCTCTGACTTCAACAGCGACAC  ACACTCCAGCTGGGTAGCTTATCAGACTGA  AACCCTGCTCGCAGTATTTGAG  TGTGCAAATCCATGCAAAACTGA  GGGGTAGCACCATTTGAA  ACACTCCAGCTGGGGTGACATCACATA  CGATCATGATGGGCTCCTCG  CTCGCTTCGGCAGCACA | TCAGGCATTTTCCCGTCC  CCTTATCAAGATGCGAACTCACA  TGGTATGTTTGTACGTCTCCAGA  AGCAGGCTTGCTTCTGACAT  GCACTGGGAGCACATTTGGA  CACCCTGTTGCTGTAGCCAAATTC  TGGTGTCGTGGAGTCG  GCGGCAGTATGGCTTTTTATTCC  GTGCAGGGTCCGAGGT  TGCGTGTCGTGGAGTC  TGGTGTCGTGG AGTCG  GTGCAGGGTCCGAGGT  AACGCTTCACGAATTTGCGT |
